# Prognosis of ER-positive, HER2-negative postmenopausal early breast cancer patients based on response to neoadjuvant endocrine therapy and multigene assay results: findings from the NEOS trial

**DOI:** 10.1007/s12282-026-01862-3

**Published:** 2026-06-18

**Authors:** Hiroji Iwata, Norikazu Masuda, Yutaka Yamamoto, Tomomi Fujisawa, Tatsuya Toyama, Masahiro Kashiwaba, Shoichiro Ohtani, Naruto Taira, Takehiko Sakai, Yoshie Hasegawa, Rikiya Nakamura, Takuhiro Yamaguchi, Kentaro Sakamaki, Christy A. Russell, Naoko Sugiyama

**Affiliations:** 1 Department of Advanced Clinical Research and Development, Nagoya City University Graduate School of Medical Sciences, 1 Aza-Kawasumi, Mizuho-Cho, Mizuho-Ku, Nagoya City, Aichi Prefecture 467-8601 Japan; 2 Department of Breast Surgery, Graduate School of Medicine, Kyoto University, 54 Shōgoin Kawaracho, Sakyo-Ku, Kyoto City, Kyoto Prefecture 606-8507 Japan; 3 Department of Breast and Endocrine Surgery, Kumamoto University Hospital, 1-1-1 Honjo, Chuo-Ku, Kumamoto City, Kumamoto Prefecture 860-8556 Japan; 4 Department of Breast Oncology, Gunma Prefectural Cancer Center, 617-1, Takabayashi Nishimachi, Ota City, Gunma Prefecture 373-8550 Japan; 5 Department of Breast Surgery, Nagoya City University, Graduate School of Medical Sciences, 1 Aza-Kawasumi, Mizuho-Cho, Mizuho-Ku, Nagoya City, Aichi Prefecture 467-8601 Japan; 6 Breast Surgery Center, Heartlife Hospital, 208 Iju, Nakagusuku Village, Nakagami District, Okinawa, Okinawa Prefecture 901-2492 Japan; 7 Ohtani Shoichiro Breast Clinic, 12-5-1 Otemachi, Naka-Ku, Hiroshima City, Hiroshima Prefecture 730-0051 Japan; 8 Breast and Thyroid Surgery, Kawasaki Medical School, 577 Matsushima, Kurashiki City, Okayama Prefecture 701-0192 Japan; 9 Breast Oncology Center, Cancer Institute Hospital, Japanese Foundation for Cancer Research, 3-8-31 Ariake, Koto-Ku, Tokyo, 135-8550 Japan; 10 Department of Breast Surgery, Hachinohe City Hospital, 3-1-1, Tamukai, Hachinohe City, Aomori Prefecture 031-8555 Japan; 11 Division of Breast Surgery, Chiba Cancer Center, 666-2 Nito-Machi, Chuo-Ku, Chiba City, Chiba Prefecture 260-8717 Japan; 12 Division of Biostatistics, Tohoku University Graduate School of Medicine, 1-1 Seiryo-Cho, Aoba-Ku, Sendai City, Miyagi Prefecture 980-8574 Japan; 13 Faculty of Health Data Science, Juntendo University, 6-8-1 Hinode, Urayasu City, Chiba Prefecture 279-0013 Japan; 14 US Medical Affairs, Exact Sciences Corporation, 301 Penobscot Drive, Redwood City, CA 94063 USA; 15 Medical Affairs, Exact Sciences K.K. Toranomon Hills Mori Tower 21F, 1-23-1 Toranomon, Minato-Ku, Tokyo, 105-0001 Japan

**Keywords:** Neoadjuvant endocrine therapy (NET), Oncotype DX, Postmenopausal breast cancer, ER positive, HER2 negative

## Abstract

**Background:**

We previously reported the 21-gene Oncotype DX® assay results from TransNEOS in patients enrolled in the phase 3 NEOS trial. However, the association between assay results and long-term prognosis has remained unclear.

**Methods:**

Of the 296 patients registered in TransNEOS, 226 patients were enrolled in this study. Multigene assay results were categorized into three groups based on Recurrence Score® (RS): RS low < 11, RS intermediate 11–25, RS high > 25. Kaplan–Meier methods evaluated the association between RS results and DDFS and OS across treatments and clinical response to neoadjuvant endocrine treatment (NET).

**Results:**

The clinical efficacy of NET was judged as CR, PR, and SD in 4 (1.8%), 113 (50%), and 109 (48.2%) patients, respectively. In the RS low and intermediate groups, no statistically significant difference in DDFS or OS was observed between the endocrine therapy (ET) alone group and the chemoendocrine therapy (CT + ET) group. In the RS high group, OS was significantly lower in the ET group compared to the CT + ET group (p = 0.037). Among patients in the RS high group who achieved CR + PR response to NET, there was no significant difference in OS between the CT + ET group and the ET alone group (p = 0.25). However, the number of events was limited, and the study may have been underpowered to detect a meaningful difference.

**Conclusions:**

The need for chemotherapy in postmenopausal HR + , HER2- breast cancer patients might be further informed by integrating the results of the Oncotype DX test with the response to NET.

**Supplementary Information:**

The online version contains supplementary material available at https://doi.org/10.1007/s12282-026-01862-3.

## Introduction

Breast cancer (BC) is one of the most commonly diagnosed cancers worldwide [1]. Hormone receptor-positive (HR +), human epidermal growth factor receptor 2 (HER2)-negative disease accounts for more than 70% of incident BC [2]. The conclusions of recent clinical trials recommend the use of CDK4/6 inhibitor [3, 4] or S-1 [5] in combination with endocrine therapy (ET) in post-operative patients with intermediate or high risk HR + , HER2- BC. The need for chemotherapy (CT) in patients with estrogen receptor (ER) + , HER2- early postmenopausal BC is determined on the basis of pathological findings such as invasive tumor size and lymph node status, in addition to the results of multigene assays using either core biopsy or surgical excision specimens. In HR + , HER2- early BC, preoperative CT is not typically recommended; however, neoadjuvant CT plus anti-HER2-directed therapy or preoperative CT with immune checkpoint blockade is often recommended for HER2 + or triple negative (TN) BC, respectively [6]. Additionally, it should be noted that pathologic complete response (pCR) rates following neoadjuvant therapy for HR + , HER2- BC are much lower than those observed for HER2 + or TN BC, and pCR has not been shown to correlate with long-term prognosis [7, 8].

A Ki67 reduction at 2 weeks with neoadjuvant ET (NET) correlates with prognosis [9], but there is no evidence to predict benefit from CT. Therefore, NET is typically not recommended in practice.

The 21-gene Oncotype DX® multigene assay, validated in several large clinical trials, is recommended for postmenopausal patients with 0 to 3 lymph node metastases and low and intermediate Recurrence Score® (RS) results, who derive no additional benefit from CT [6, 10–12].

We had previously reported on a clinical trial (NEOS study) to test whether the need for CT can be determined based on the therapeutic effect of NET. The results concluded that it is difficult to determine the need for CT based solely on the effect of NET [13]. Furthermore, in the TransNEOS study, we had reported that the Oncotype DX test was performed by needle biopsy in T1c (2 cm) and T2N0 cases from patients enrolled in the NEOS trial and that it can predict the efficacy of NET [14]. In the present study, additional analyses were conducted on the results of TransNEOS and the relationship between the effect of NET and long-term prognosis in the patients who underwent Oncotype DX testing.

## Patients and methods

### Study patients

Patients eligible for the TransNEOS study were previously enrolled in the parent NEOS study and were recruited from the 25 NEOS study centers with the highest patient enrollment [14]. Eligible patients provided informed consent. They were postmenopausal women < 75 years of age with T1cT2 (≥ 2 cm in the largest dimension, as measured by magnetic resonance imaging [MRI] and/or computed tomography scan), clinically node-negative, non-metastatic, ER + , HER2- invasive BC who received neoadjuvant letrozole. Core-biopsy samples were obtained from eligible patients before neoadjuvant treatment and were later sent for Oncotype DX testing (Genomic Health, Inc., Redwood City, CA). Patients with no RS results, no tumor block available, insufficient tumor as determined by a pathologist at Genomic Health in accordance with standard operating procedures, or insufficient or inadequate RNA for testing were excluded. Unique patient and specimen identifiers were assigned by the principal investigator and anonymized for Genomic Health staff.

### Study protocol

All patients included in the TransNEOS study received 24–28 weeks of neoadjuvant letrozole per the NEOS study protocol. Oncotype DX testing was performed in a central laboratory (Genomic Health, Inc., Redwood City, CA) on RNA extracted from formalin-fixed paraffin-embedded core-biopsy samples, as previously described [14].

Distant Disease-Free Survival (DDFS) was defined as the time to recurrence in distant organs such as the bone, liver, lung, and brain, excluding soft tissue or locoregional disease, and death from any cause since randomization. Overall survival (OS) was defined as the time from the date of primary enrollment until the date of death from any cause. Clinical staging, histological classification, and clinical response to neoadjuvant ET were assessed in accordance with the General Rules for Clinical and Pathological Recording of Breast Cancer 15th Edition [15]. The Japanese version of the Eastern Cooperative Oncology Group scale was also used to grade performance status. Clinical response was evaluated by inspection/palpation and ultrasonography (US) at each specified time point, and computed tomography or MRI was performed at the completion of NET. The clinical response evaluation was only performed for the primary tumor, and not for the lymph node area. Complete clinical response (CR), clinical partial response (PR), and clinical stable disease (SD) were defined as the complete disappearance, a ≥ 30% reduction, and changes ranging from a reduction of up to 30% to an increase of less than 20% in the primary tumor on imaging at the completion of NET, respectively. ER, PgR, and HER2 status and nuclear grade as eligibility criteria were assessed by local pathologists. Standard RS result risk categories were used as follows: RS low < 11, RS intermediate 11–25, and RS high > 25. Both local and central pathology determinations of ER (immunohistochemistry [IHC] or Allred score), PgR (IHC or Allred score), and HER2 (IHC and/or fluorescent in situ hybridization), and central pathology determination of Ki67 (IHC) were performed on all tumor samples using standard techniques.

### Statistical analysis

The present analysis was exploratory in nature. Kaplan–Meier methods were used to estimate DDFS and OS according to RS group and clinical response to NET. Survival curves were compared using the log-rank test. All hypothesis tests were reported using two-sided p-values. HRs and 95% CIs were estimated with Cox proportional hazards models when events occurred in both groups. In subgroups with no events in one arm, HRs were not estimable, and results were described accordingly. All analyses were performed using R version 4.4.3 (R Foundation for Statistical Computing, Vienna, Austria).

## Results

**Fig. 1:**
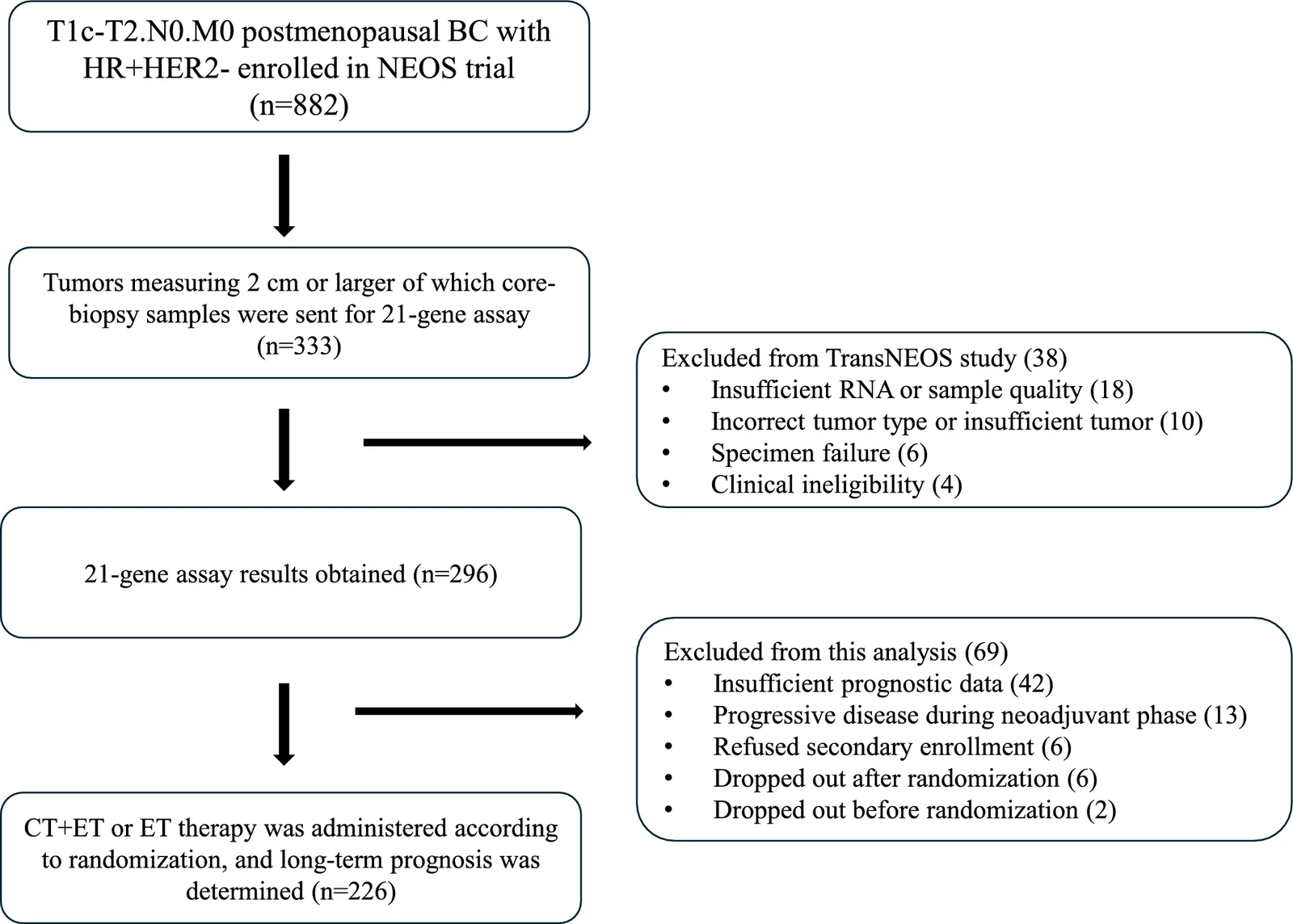
Consort diagram of this study

In total, 333 core-biopsy samples were submitted for Oncotype DX testing. Of these, 38 samples were excluded due to insufficient RNA or sample quality (n = 18), incorrect tumor type or insufficient tumor (n = 10), specimen failure (n = 6), and clinical ineligibility (n = 4). Of the remaining 295 samples, 226 were analysed. Patients were excluded if they had progressive disease during neoadjuvant therapy (13), refused secondary enrollment (6), dropped out before/after randomization (8), or if there was insufficient prognostic data (42) (Fig. 1). The median follow-up was 7.8 years (range 0.1–19.6 years). Characteristics of this analysis cohort (N = 226) are summarized in Table 1. Median age was 63 (49–75) years, mean tumor size was 26.6 mm, the majority of patients had nuclear grade 1 disease (158); ER, PgR, and HER2 statuses were determined by RT-PCR and Ki67 status was determined by IHC. PgR positive/negative in 175/51 patients; HER2 positive in 7 patients; Ki67 < 10%, 10–30%, and > 30 in 66, 96, and 44 patients, respectively. Most patients (54.9%) had an RS intermediate result, while 27.4% had a RS low result and 17.7% had a RS high result.

**Table 1:** Patient characteristics

	Total 226 patients	%
Age		
≦60	62	29.5
60 ~ 70	123	58.6
> 70	25	11.9
Clinical T size		
2 cm	38	17.0
> 2 cm	186	83.0
Nuclear grade		
1	158	72.2
2	42	19.2
3	19	8.7
ER status by RT-PCR		
Postive	226	100
Negative	0	0
PgR status by RT-PCR		
Positive	175	77.4
Negative	51	22.6
HER2 status by RT-PCR		
Negative	177	78.3
Equivocal	42	18.6
Positive	7	3.1
Ki67 by IHC		
< 10%	66	32.0
10 ~ 30%	96	46.6
> 30%	44	21.4
RS		
< 11	62	27.4
11 ~ 25	124	54.9
> 25	40	17.7
NET response		
CR	4	1.8
PR	113	50.0
SD	109	48.2

*ER* Estrogen receptor, *HER2* Human epidermal growth factor receptor 2, *IHC* Immunohistochemistry, *PgR* Progesterone receptor, *RT-PCR* Reverse transcription-polymerase chain reaction, *NET* Neoadjuvant endocrine therapy, *CR* Complete response, *PR* Partial response, *SD* Stable disease, *RS* Recurrence score®

**Fig. 2:**
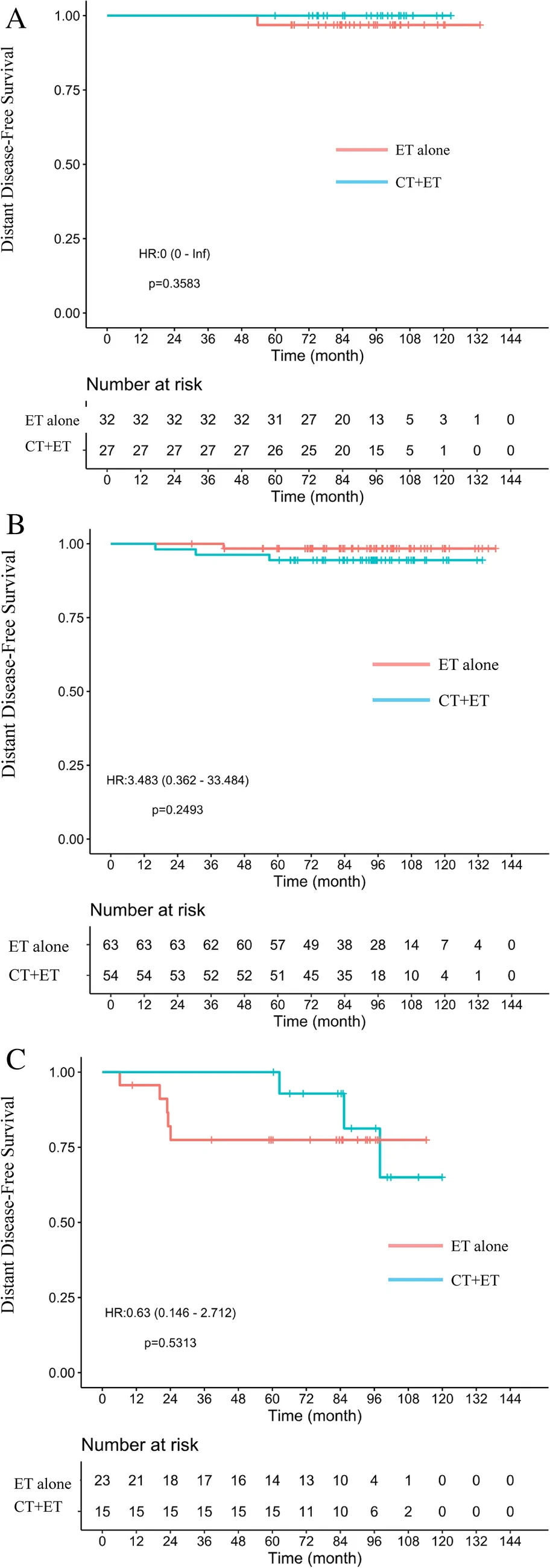
Distant Disease-Free Survival (DDFS) in RS groups treated with ET alone and with CT + ET. HR: NE (95% CI, NE–NE), due to absence of events in one group. **A** RS low group (p = 0.3583), **B** RS intermediate group (p = 0.2493), **C** RS high group (p = 0.5313); *RS* Recurrence score, *ET* Endocrine therapy, *CT* Chemotherapy

**Fig. 3:**
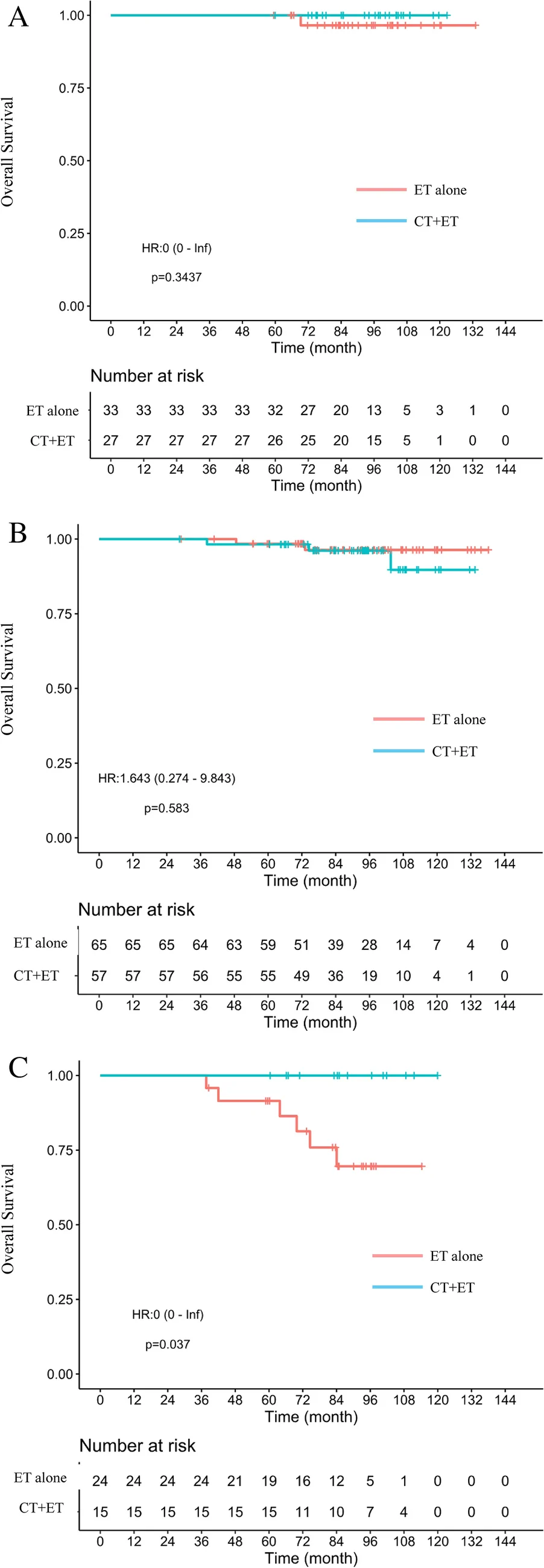
Overall Survival (OS) in RS groups treated with ET alone and with CT + ET. HR: NE (95% CI, NE–NE), due to absence of events in one group. **A** RS low group (p = 0.3437), **B** RS intermediate group (p = 0.583), **C** RS high group (p = 0.037); *RS* Recurrence score, *ET* Endocrine therapy, *CT* Chemotherapy

The clinical efficacy of NET was judged to be CR, PR, and SD in 4 (1.8%), 113 (50%), and 109 (48.2%) patients, respectively. Prognosis by each RS result group showed that the RS low group only had one DDFS event in the ET alone group, with no statistically significant difference between the CT + ET group and the ET alone group (Fig. 2A, p = 0.35). In the RS intermediate group, one DDFS event was found in the ET alone group and three events in the CT + ET group (Supplementary Table 1), with no statistically significant difference (Fig. 2B, p = 0.24). In the RS high group, the ET alone group had early DDFS events but no recurrence after 24 months, whereas the CT + ET group had recurrent events after 60 months instead of fewer early recurrences (Fig. 2C, HR: 0.63 (0.146–2.712), p = 0.53) (Supplementary Table 1). For the OS endpoint, one death was observed in the RS low group treated with ET alone (Fig. 3A) (Supplementary Table 2). In the intermediate group, there was no statistically significant difference between the two treatment groups (Fig. 3B, HR: 1.64 (0.27–9.84), p = 0.583). In the RS high group, the prognosis tended to be worse in the ET alone group, with no deaths in the CT + ET group at a median observation period of 7.8 years (Fig. 3C, p = 0.037) (Supplementary Table 2). However, the number of events was limited, and the study may have been underpowered to detect a meaningful difference if it exists.

**Fig. 4:**
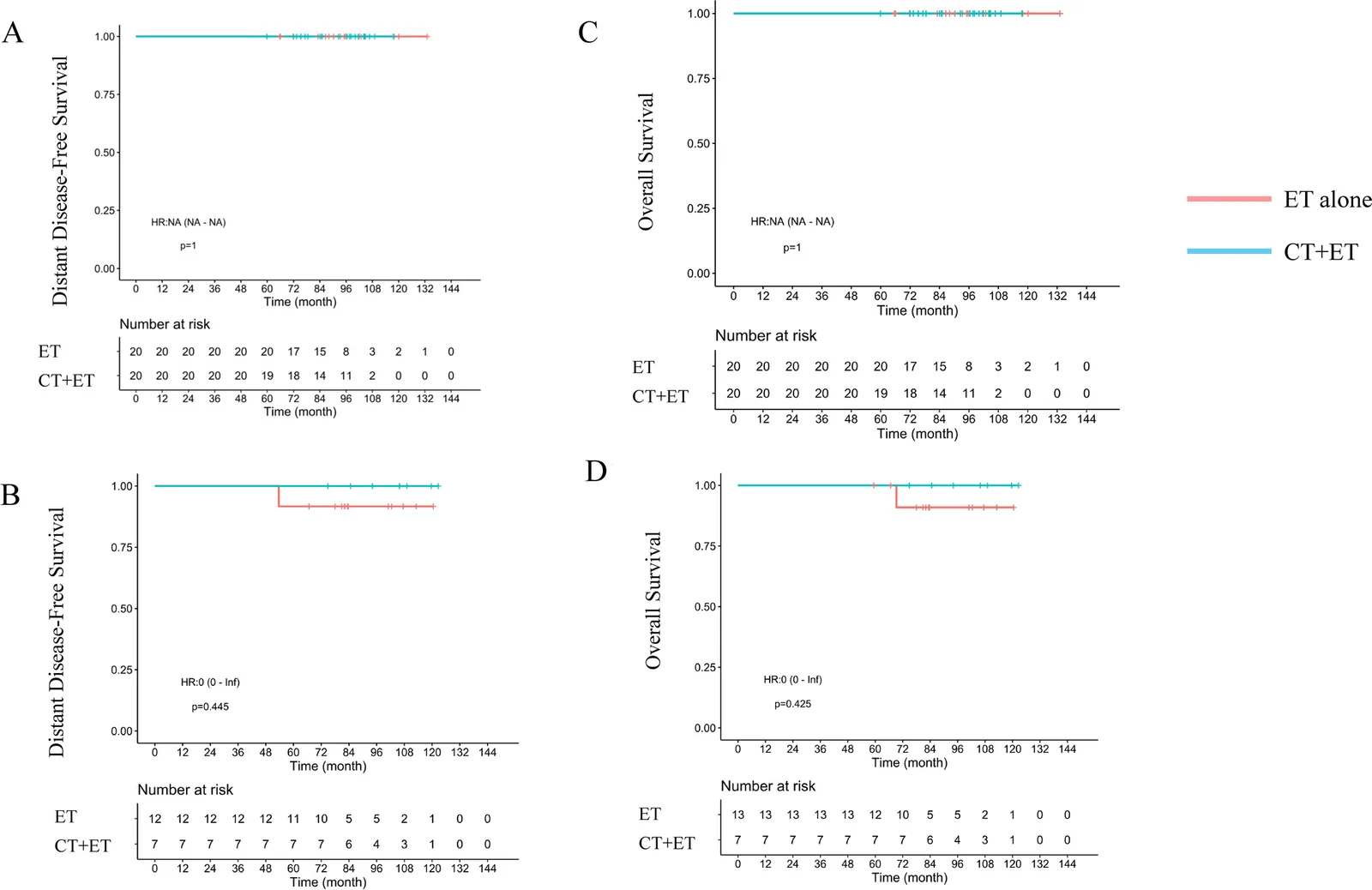
Distant disease-free survival (DDFS) and overall survival (OS) in RS low group treated with ET alone and with CT + ET. HR: NE (95% CI, NE–NE), due to absence of events in one group. **A** DDFS in CR + PR group (p = 1), **B** DDFS in SD group (p = 0.445), **C** OS in CR + PR group (P = 1), **D** OS in SD group (p = 0.425)

**Fig. 5:**
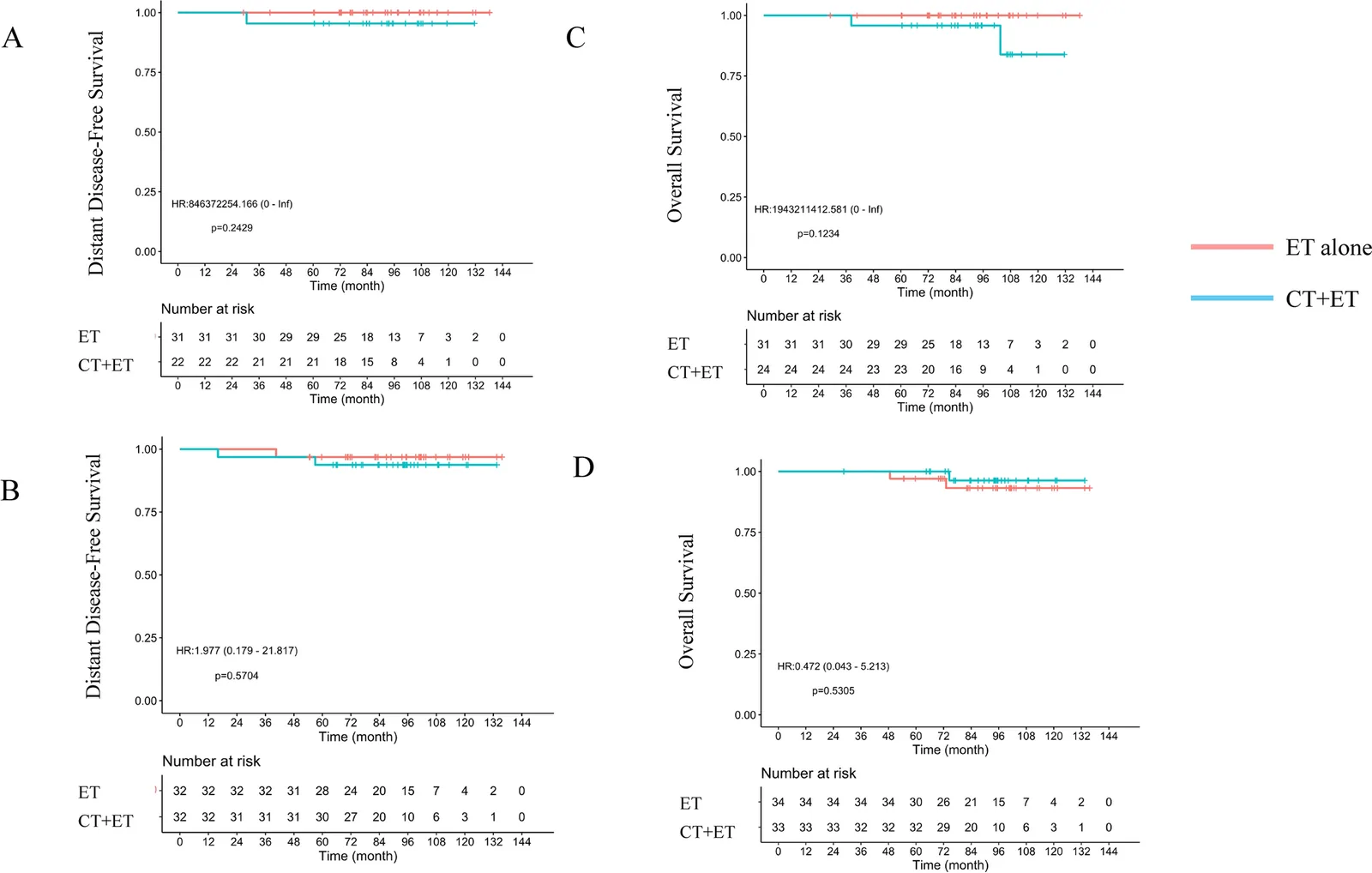
Distant disease-free survival (DDFS) and overall survival (OS) in RS intermediate group treated with ET alone and with CT + ET. HR: NE (95% CI, NE–NE), due to absence of events in one group. **A** DDFS in CR + PR group (p = 0.2429), **B** DDFS in SD group (p = 0.5704), **C** OS in CR + PR group (p = 0.1234), **D** OS in SD group (p = 0.5305)

**Fig. 6:**
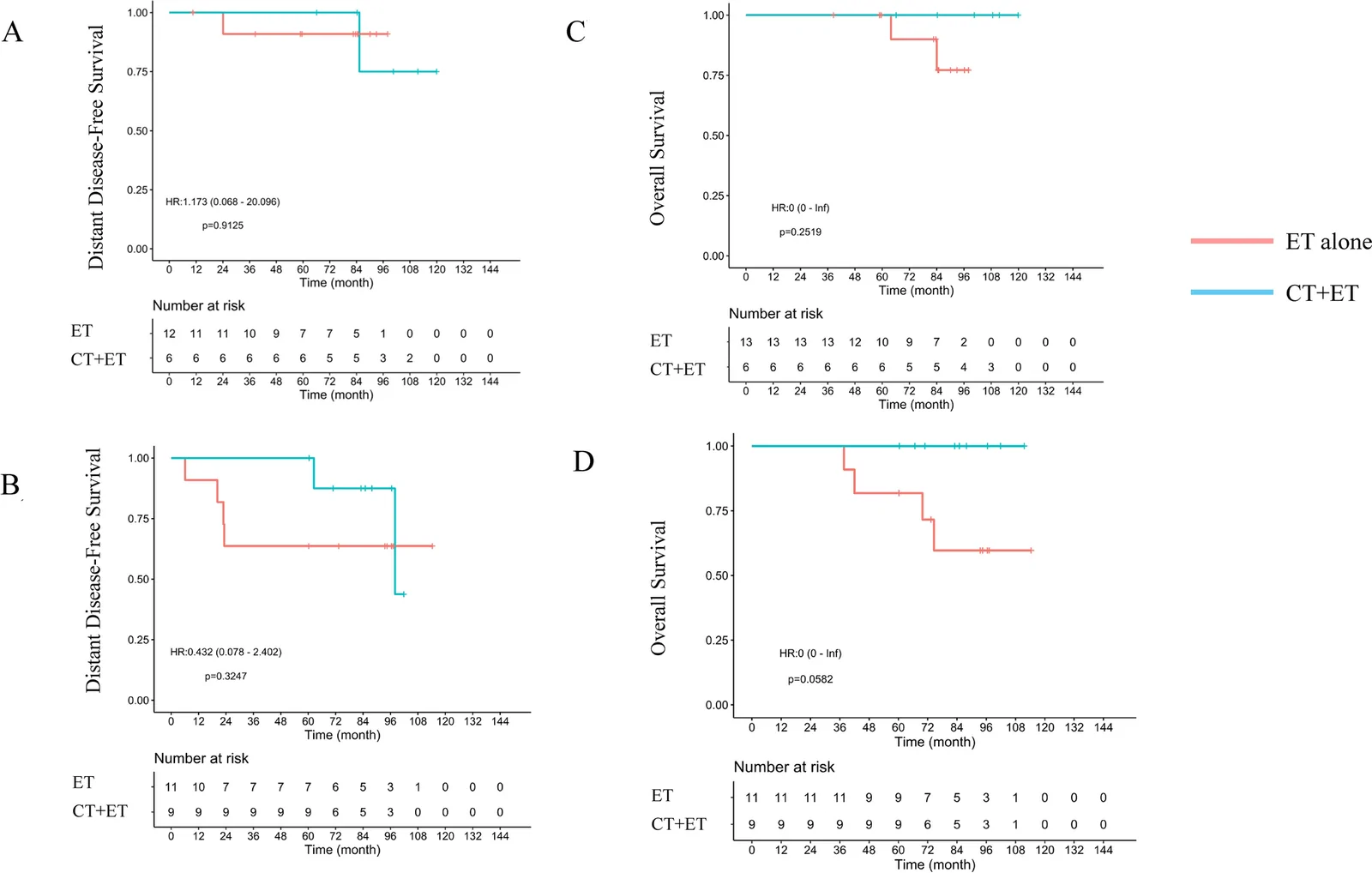
Distant disease-free survival (DDFS) and overall survival (OS) in RS high group treated with ET alone and with CT + ET. HR: NE (95% CI, NE–NE), due to absence of events in one group. **A** DDFS in CR + PR group (p = 0.9125), **B** DDFS in SD group (p = 0.3247), **C** OS in CR + PR group (p = 0.2519), **D** OS in SD group (p = 0.0582)

When looking at the effect of CT add-on in the CR + PR and SD groups in the ET response by each RS result group, the RS low group had fewer overall recurrent events, and there was no statistically significant difference between the CT + ET group and ET alone group in either of the CR + PR or SD groups (Fig. 4A–D). In the RS intermediate group, there were also no statistically significant differences between the CT + ET group and ET alone group, in either of the CR + PR or SD groups (Fig. 5A–D). In the RS high group, the CR + PR group showed no statistically significant difference in either DDFS or OS between the CT + ET and ET alone groups (Fig. 6A, C), while in the SD group, the ET alone group had more cases of early distant recurrence than the CT + ET group (Fig. 6B, p = 0.32) (Supplementary Tables 1 and 2) and tended to have worse survival rates (Fig. 6D, P = 0.0582).

## Discussion

The clinical efficacy of preoperative ET combined with RS result by the Oncotype DX® assay was used to determine whether the addition of CT is worthwhile in ER + , HER2- postmenopausal BC patients.

For postmenopausal BC patients with tumors ≥ 2 cm in diameter and a RS high result > 25, postoperative chemotherapy is recommended [6]. In this trial, consistent with previous reports, recurrence events were fewer in cases with an RS result of 25 or below, and no improvement in prognosis was demonstrated by adding chemotherapy. In the group with an RS greater than 25, no difference was observed with the addition of chemotherapy in the subgroup that responded to NET (CR + PR); however, due to the small number of patients and events, no definitive conclusion could be drawn.

In previous TransNEOS studies, legacy cutpoints of RS results (< 18, 18—30, > 30) were used. However, in the present study, considering the acquisition of data with broader clinical applicability, analysis was performed using cutpoint values (< 11, 11—25, > 25) employed in the TAILORx trial [12], which are standard in clinical practice. The distribution of RS low, intermediate, and high results in the present study was 27.4%, 54.9%, and 17.7%, respectively. Compared to the TAILORx trial [12], which used the same cutoff values (17%, 69%, and 14%), the proportion of RS low results was higher. Furthermore, in the SAFIA clinical trial [16], which included patients with positive lymph node metastasis, the distribution was 13.3%, 55.9%, and 30.3%, showing a higher proportion of RS high results. The higher proportion of RS low results in the present study is thought to be due to the restriction to postmenopausal BC and the inclusion only of patients clinically classified as lymph node negative.

The current standard is to proceed to primary surgery in lieu of neoadjuvant systemic therapy in postmenopausal ER + patients with clinically negative lymph nodes or with a low lymph node burden (< 3 lymph nodes positive). If the Oncotype DX test is performed on the surgical specimen and the patient has RS low or intermediate results, CT omission is the standard of care [6, 12].

The present study also found no benefit of additional CT in the RS low or intermediate groups, regardless of the effect of NET, excluding patients who progressed during NET. This is consistent with the results of clinical trials involving postmenopausal breast cancer patients with 0 to 3 metastatic lymph nodes post-surgery, in whom the addition of chemotherapy is not effective [11, 12]. Furthermore, the finding that adding chemotherapy to the RS high group improves prognosis is consistent with existing data [10, 17]. However, even within the RS high group, the number of events was very low among patients who achieved CR or PR following NET, regardless of whether chemotherapy was added. The statistical power is limited, making it difficult to draw conclusions regarding the need for chemotherapy based on the assessment of NET efficacy. TransNEOS results indicate that the RS high group responded poorly to NET [14]. In the TransNEOS trial, 22% of patients with RS results > 30 achieved CR + PR [14]. Further data may help to clarify whether chemotherapy is necessary in the CR + PR subgroup of the RS-high group.

The ADAPT trial demonstrated that an early decrease in Ki67 after the initiation of neoadjuvant endocrine therapy is associated with survival outcomes, highlighting the potential importance of biological response in evaluating NET efficacy [18]. In contrast, the TransNEOS trial did not assess changes in Ki67, and response to NET was primarily evaluated based on clinical tumor shrinkage. Therefore, the interpretation of NET response may extend beyond clinical assessment alone and may benefit from incorporating biological markers that reflect tumor proliferation dynamics. A Japanese group is also planning a clinical trial to determine the need for post-operative CT in pre-menopausal patients by assessing the combined effects of NET and evaluation by the Oncotype DX test (data not published).

NET is an excellent tool for investigating individual patient response to treatment and is expected to be a therapeutic tool combined with multigene assay to determine the need for CT after surgery.

A limitation of this study is that the parent trial enrolled 888 cases, but only 226 cases reached prognostic analysis in TransNEOS, which is a limited population analysis. The overall number of distant recurrence and death events was small, making robust statistical analysis difficult. Therefore, the study may have been underpowered to detect a meaningful difference. An ideal analysis would be in the setting of an exploratory prospective-retrospective randomized controlled trial. The primary endpoint of the NEOS trial was DFS, not OS. Furthermore, the number of events did not reach the planned sample size, resulting in reduced statistical power [13]. The TransNEOS study was conducted in patients from the NEOS trial who had tumors measuring 2 cm or larger and who were eligible for long-term follow-up [14]. Consequently, the number of events and patients at risk was low in this all analyses, and caution is required when interpreting the statistical results. The change in Ki67 expression at 2 weeks after treatment initiation is considered a predictive factor for the efficacy of NET and a long-term outcome [9]. However, because the NEOS trial design did not include Ki67 measurement at 2 weeks after treatment initiation, this study could not examine the relationship between the change in Ki67 expression, RS results and the efficacy of NET.

This study was a retrospective analysis using samples from a prospective randomized trial. Although it has several limitations, it represents the first attempt to comprehensively evaluate prognosis by integrating results from a multigene assay with the effects of NET.

## Conclusion

The need for chemotherapy in postmenopausal HR + , HER2- breast cancer patients might be further informed by integrating the results of the Oncotype DX test with the response to NET.

## Supplementary Information

Below is the link to the electronic supplementary material.Supplementary file1 (DOCX 20 KB)

## Data Availability

The data that support the findings of this study are available from the corresponding author upon reasonable request.
