# Supplementary material for: Prognosis of ER-positive, HER2-negative postmenopausal early breast cancer patients based on response to neoadjuvant endocrine therapy and multigene assay results: findings from the NEOS trial
Source: Breast Cancer. 2026 Jun 18;33(5):1004–13. doi: 10.1007/s12282-026-01862-3 (PMC13522011; doi:10.1007/s12282-026-01862-3)
Supplement: Supplementary file 1 [file 12282_2026_1862_MOESM1_ESM.docx]

Supplementary Table1 : Cumulative DDFS events at time points

| RS status | Regimen | Time (M) | Patients  at Risk (n) | Cumulative  Events (n) |
| --- | --- | --- | --- | --- |
| low | ET alone | 60 | 31 | 1 |
|  |  | 96 | 13 | 1 |
|  | CT + ET | 60 | 26 | 0 |
|  |  | 96 | 15 | 0 |
| intermediate | ET alone | 60 | 57 | 1 |
|  |  | 96 | 28 | 1 |
|  | CT + ET | 60 | 51 | 3 |
|  |  | 96 | 18 | 3 |
| high | ET alone | 60 | 14 | 5 |
|  |  | 96 | 4 | 5 |
|  | CT + ET | 60 | 15 | 0 |
|  |  | 96 | 6 | 2 |

DDFS: Distant Disease Free Survival, RS: Recurrence Score^®^, ET: Endocrine therapy,

CT: Chemothrerapy, M: Months

Supplementary Table 2: Cumulative OS events at time points

| RS status | Regimen | Time (M) | Patients  at Risk (n) | Cumulative  Events (n) |
| --- | --- | --- | --- | --- |
| low | ET alone | 60 | 32 | 0 |
|  |  | 96 | 13 | 1 |
|  | CT + ET | 60 | 26 | 0 |
|  |  | 96 | 15 | 0 |
| intermediate | ET alone | 60 | 59 | 1 |
|  |  | 96 | 28 | 2 |
|  | CT + ET | 60 | 55 | 1 |
|  |  | 96 | 19 | 2 |
| high | ET alone | 60 | 19 | 2 |
|  |  | 96 | 5 | 6 |
|  | CT + ET | 60 | 15 | 0 |
|  |  | 96 | 7 | 0 |

OS: Overall Survival, RS: Recurrence Score^®^, ET: Endocrine therapy,

CT: Chemothrerapy, M: Months
